# Efficacy and Safety of Ningmitai Capsules in Patients with Chronic Epididymitis: A Prospective, Parallel Randomized Controlled Clinical Trial

**DOI:** 10.1155/2021/9752592

**Published:** 2021-03-27

**Authors:** Zhang Jing, Guan Liying, Wang Zhenqing, Zhang Hui, Liu Shuai, Sun Dingqi, Fu Qiang, Zhang Keqin

**Affiliations:** ^1^Department of Nephrology, Shandong Provincial Hospital Affiliated to Shandong First Medical University, Jinan 250021, Shandong, China; ^2^Physical Examination Center, Shandong Provincial Hospital Affiliated to Shandong First Medical University, Jinan 250021, Shandong, China; ^3^Department of Urology, Shandong Provincial Hospital Affiliated to Shandong First Medical University, Jinan 250021, Shandong, China

## Abstract

**Objectives:**

To evaluate the efficacy and safety of Ningmitai (NMT) capsules in patients with chronic epididymitis.

**Methods:**

This prospective randomized controlled trial included 112 patients diagnosed with chronic epididymitis. The patients were randomized (1 : 1 : 1) to receive levofloxacin (LVX), NMT, or NMT combined with LVX for 4 weeks. The patients were followed up at 2 and 4 weeks after initiation of treatment and were evaluated in terms of Chronic Epididymitis Symptom Index (CESI) scores, epididymal nodules, and safety parameters. The primary endpoints were the CESI scores at the end of 2 and 4 weeks of treatment. The secondary endpoints included the mean epididymal nodule diameter and the clinical efficacy rate. Safety was evaluated by hepatorenal function tests and adverse event reports during the trial.

**Results:**

After 2 weeks of treatment, the CESI score of the NMT group was significantly lower than that of the LVX group (*P* < 0.05). In addition, the clinical efficacy rate of the NMT group was significantly higher than that of the LVX group (55% vs. 8.33%, *P* < 0.0001), indicating that NMT has a rapid effect on chronic epididymitis. After 4 weeks of treatment, there was no significant difference in CESI scores or clinical efficacy rates between the two monotherapy regimens (*P* > 0.05); however, the mean diameter of epididymal nodules was significantly smaller in the NMT group than in the LVX group (*P* < 0.0001). Moreover, after 4 weeks of treatment, the patients in the LVX + NMT group, which had a clinical efficacy rate of 97.22%, had lower CESI scores (both *P* < 0.01) and a smaller epididymal nodule diameter (vs. LVX, *P* < 0.0001; vs. NMT, *P* < 0.05) than those in the other two groups. No adverse events or abnormal hepatorenal function were found during the study.

**Conclusion:**

NMT significantly improved CESI scores and epididymal nodule diameter in patients with chronic epididymitis. The combination of NMT and LVX provides a much better effect than monotherapy, and this treatment regimen was well tolerated.

## 1. Introduction

Chronic epididymitis is a common disease of the male reproductive system. The aetiology of chronic epididymitis is complicated and varied, and the pathogenesis of the disease is usually identified as secondary to prostatitis or urinary tract infections [1]. There is no standard therapy for chronic epididymitis, and the administration of antibiotics remains the most common treatment [2].

Increasing evidence has demonstrated that complementary and alternative medicine is important and effective in the management of different chronic diseases, including urinary retention [3], degenerative knee osteoarthritis [4], gastroesophageal reflux disease [5], chronic idiopathic urticaria [6], and shigellosis [7].

Ningmitai (NMT) capsules, a formulated Chinese medicine, are composed of Touhualiao (*Polygonum capitatum* Buch.-Ham. ex D. Don), Baimaogen (*Imperata cylindrica* Beauv. var. *major* (Nees) C. E. Hubb.), Dafengteng (*Cocculus orbiculatus* (L.) DC.), Sankezhen (*Berberis soulieana* Schneid., *Berberis wilsonae* Hemsl., *Berberis poiretii* Schneid., and *Berberis vernae* Schneid.), Xianhecao (*Agrimonia pilosa* Ledeb.), Mufurongye (*Hibiscus mutabilis* L.), and Lianqiao (*Forsythia suspensa* (Thunb.) Vahl) [8]. These herbal components contribute to multiple pharmacological effects of NMT, including its antibacterial, anti-inflammatory, and analgesic actions, among others [9, 10]. NMT has been widely used for decades in the treatment of genitourinary diseases, including chronic prostatitis and lower urinary tract infection [11–13] (approval number from the National Medical Products Administration of China: Z20025442). A previous study indicated that NMT in combination with antibiotics had a beneficial clinical effect in the treatment of acute epididymitis [14]. However, the efficacy of NMT for chronic epididymitis remains unclear. Therefore, a prospective randomized study was conducted to assess the efficacy and safety of NMT alone or in combination with antibiotics in patients with chronic epididymitis.

## 2. Methods

### 2.1. Study Design

This was a prospective, parallel randomized controlled study conducted at the Department of Urology, Shandong Provincial Hospital. Patients diagnosed with chronic epididymitis from June 2017 to March 2019 were screened, and the eligible patients were randomly (1 : 1 : 1) divided into three groups—a levofloxacin (LVX) group, an NMT group, and a combined (LVX + NMT) group—according to a random code sequence generated by SPSS 18.0 software. In the LVX group, patients received LVX (Daiichi Sankyo Pharmaceutical Co., Ltd. Beijing, China) 0.5 g q.d. in the morning for 4 weeks. In the NMT group, patients received NMT (Guiyang Xin Tian Pharmaceutical Co., Ltd., China) 0.38 g × 4 capsules t.i.d. for 4 weeks. Each NMT capsule contains no less than 1 mg gallic acid according to the national drug standard WS-10348- (ZD-0348) 2002-2012Z and the Chinese pharmacopoeia. In the LVX + NMT group, patients received LVX (0.5 g q.d. in the morning) plus NMT (0.38 g × 4 capsules t.i.d.) for 4 weeks.

This study was conducted under Good Clinical Practice requirements and approved by the Ethics Committee of Shandong Provincial Hospital affiliated with Shandong University, China (No. 2017-054). All patients provided written informed consent before any study procedures.

### 2.2. Patients

Patients receiving LVX and/or NMT were required to meet the following criteria to be included in this study [15]: (1) age ranging from 18 to 60 years; (2) a history of acute epididymitis or chronic prostatitis; (3) discomfort such as swelling and/or pain that occurred in one or both epididymides or part of the scrotum; (4) mild, intermittent discomfort to severe, persistent pain, and other symptoms of varying degrees; (5) unilateral or bilateral enlargement of the epididymis, with epididymal nodules; (6) local nodular enlargement, irregular margins with low or slightly strong echoes, and abundant blood flow signal on ultrasonography; and (7) willingness to participate in and complete this study.

The exclusion criteria were as follows: (1) suspected or confirmed epididymal tuberculosis; (2) epididymal or testicular tumours; (3) acute testicular epididymitis or an acute episode of chronic epididymitis; (4) severe heart, liver, kidney, or haematopoietic diseases; (5) use of similar drugs within two weeks before treatment; and (6) mental disorders or severe mental disorders.

### 2.3. Efficacy Assessments

To evaluate the severity of chronic epididymitis, Nickel [16] proposed the Chronic Epididymitis Symptom Index (CESI), which includes 2 domains: a chronic epididymitis pain subscore (range 0∼15) and a chronic epididymitis quality-of-life impact domain (range 0∼12). Because it accurately assesses the severity of chronic epididymitis, the CESI has been widely recognized and used in clinical practice and clinical research for baseline evaluation and follow-up of patients with chronic epididymitis [17–19].

The primary endpoints in this study were the mean CESI scores after 2 and 4 weeks of treatment. The secondary endpoints were the mean epididymal nodule diameter and the clinical efficacy rate. The clinical efficacy criteria were defined as follows. Effective: the CESI pain score decreased to less than 6 points or reduced by at least 3 points and symptoms improved. Ineffective: the CESI pain score was greater than 5 points or decreased by less than 3 points, and symptoms were unimproved or even worsened.

In our study, the CESI score was determined in all patients before treatment and at the end of 2 and 4 weeks of treatment. Before treatment and after 4 weeks of treatment, all patients underwent colour Doppler ultrasonography. The patients were placed in the supine position, and the penis was placed against the abdominal wall. The scrotum was fully exposed. Three diameters of epididymal nodule were measured by three experienced ultrasound doctors separately.

### 2.4. Safety Assessments

Safety assessment was based on adverse event reports and hepatorenal function tests, including the examination of alanine aminotransferase (ALT), blood urea nitrogen (BUN), and creatinine (CREA) values.

### 2.5. Statistical Analyses

All relevant data were collected and statistically analysed. The measurement data were expressed as the mean ± SD. The baseline characteristics of the study groups were analysed by one-way analysis of variance if the multiple sets of variables met the assumptions of normality and homogeneity of variance; otherwise, the Kruskal–Wallis test was used. The changes over time in the groups were analysed by two-way repeated measure analysis of variance, and the differences were then calculated by Tukey's multiple comparison test. Categorical data are reported as percentages and were compared using the *χ*^2^ test. Statistical analysis was performed using GraphPad Prism version 8.0.2 (GraphPad Software, Inc., La Jolla, CA, USA). *P* < 0.05 was considered statistically significant.

## 3. Results

### 3.1. Baseline Characteristics

**Figure 1:**
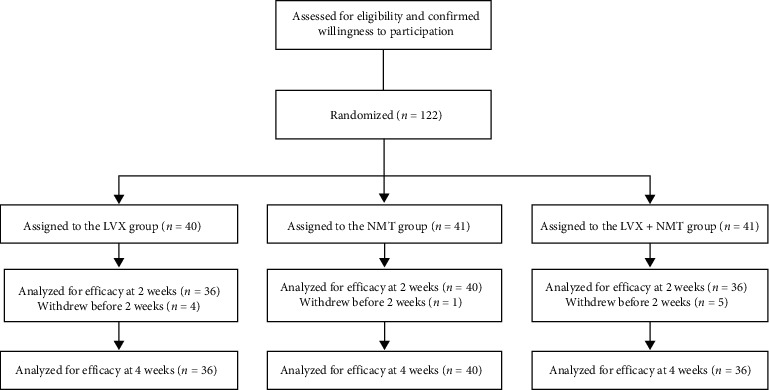
Patient flowchart.

A total of 122 patients were enrolled in the study and randomly allocated at a ratio of 1 : 1 : 1 to receive three different treatments. Ten patients withdrew during the treatment and the follow-up period; thus, 112 patients were available for the efficacy evaluation (Figure 1).

The baseline demographic and clinical characteristics of the patients are given in Table 1. There were no significant differences with respect to age, baseline CESI score, or mean diameter of epididymal nodules (MDE) among the LVX, NMT, and LVX + NMT groups (*P* > 0.05).

### 3.2. Therapeutic Effects

The posttreatment CESI scores of the three groups are summarized in Table 2. After 2 or 4 weeks of treatment, the CESI scores of all the groups had decreased significantly compared with the baseline score (*P* < 0.05), and the effect was further enhanced as the treatment time was extended (4-week vs. 2-week, *P* < 0.05). In addition, after 2 weeks of treatment, the CESI score of the NMT group was significantly lower than that of the LVX group (NMT group vs. LVX group, *P* < 0.05) and further decreased to 12.53 ± 2.64 in the combination group (LVX + NMT group vs. LVX group, *P* < 0.0001). After 4 weeks of treatment, the CESI score of the LVX + NMT group was 8.64 ± 2.15, which was lower than that of either the LVX or NMT group (both *P* < 0.01).

**Figure 2:**
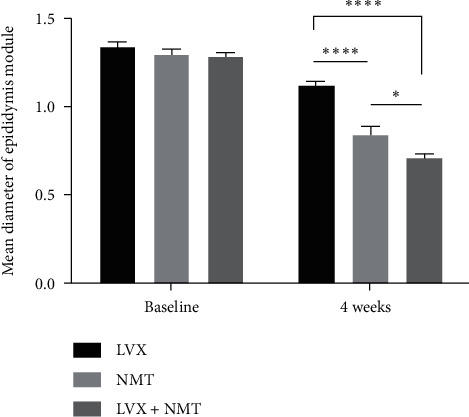
Mean diameter of epididymal nodules in the LVX group (*n* = 36), the NMT group (*n* =40), and the combined group (*n* = 36) before and after 4 weeks of treatment. Unit, cm. ^*∗*^*P* < 0.05^*∗∗∗∗*^:*P* < 0.0001. LVX, levofloxacin; NMT, Ningmitai.

As shown in Figure 2, the ultrasound results showed that after 4 weeks of treatment, the MDE was significantly smaller in the NMT group and LVX + NMT group than in the LVX group (*P* < 0.0001), which indicated that NMT, with or without LVX, may be superior to LVX alone in reducing the size of epididymal nodules.

The clinical efficacy rates after 2 or 4 weeks of treatment are given in Table 3. After 2 weeks of treatment, the clinical efficacy rates of the NMT group and the LVX + NMT group were significantly higher than those of the LVX group (NMT group vs. LVX group, 55% vs. 8.33%, *P* < 0.0001; LVX + NMT group vs. LVX group, 61.11% vs. 8.33%, *P* < 0.0001). After 4 weeks of treatment, there was a significant increase in the clinical efficacy rate of each group compared with 2 weeks of treatment (all *P* < 0.05). In particular, the effective percentage of clinical response in the LVX + NMT group was 97.22%, which was the highest rate in any group.

### 3.3. Safety Evaluation

The evaluation of hepatorenal function after 4 weeks of treatment, including ALT, BUN, and CREA, is given in Table 4. All data were within the reference value range, and no abnormal hepatorenal function was found in any of the three groups following treatment.

## 4. Discussion

The aetiology of epididymitis is complex and diverse, but its pathogenesis is usually considered to occur after prostatitis or urinary tract infection; as the disease develops, it seriously affects patients' mental health and quality of life [15]. At present, the treatment of epididymitis with extracts has not been determined. Antibiotics (74%) and anti-inflammatory drugs (36%) are still the main clinical treatments [16]. In addition, some chronic epididymitis patients choose epididymectomy in response to lingering pain, which is usually caused by varicocele leading to a decrease in local immune activity [20]. However, a previous study indicated that patients with chronic pain from epididymitis have only a 55% chance of improvement [21]. Therefore, a combination of modalities is considered beneficial for patients with chronic epididymitis [22].

In recent years, traditional Chinese medicine (TCM), as a common complementary and alternative medicine therapy, has been used in the treatment of chronic epididymitis [23]. NMT is an effective TCM product based on its multicomponent, multitarget, and multimechanism therapeutic philosophy. NMT contains certain herbals and effective constituents such as gallic acid, quercetin, rutin, berberine, and luteolin, which play important anti-inflammatory, antioxidative, analgesic, and antibacterial roles [21, 24–27]. Since the symptoms targeted by NMT are consistent with those of chronic epididymitis, we suspected that NMT might be effective against chronic epididymitis.

This was the first parallel randomized controlled clinical study to evaluate the efficacy and safety of NMT capsules in chronic epididymitis. LVX, an antibody that is commonly used in the clinic for chronic epididymitis, was chosen as a control drug. In this study, after 2 weeks of treatment, the CESI score of the NMT group was significantly lower than that of the LVX group (*P* < 0.05), and the clinical efficacy rate was significantly higher than that of the LVX group (*P* < 0.0001), indicating that NMT has rapid efficacy in chronic epididymitis. After 4 weeks of treatment, the outcomes including the CESI score, MDE, and clinical efficacy rate were all further improved in the NMT group, and the MDE of the NMT group was remarkably smaller than that of the LVX group (*P* < 0.0001). A recent research revealed that NMT has anti-inflammatory and antioxidative effects in an autoimmune prostatitis rat model and improves chronic pain by decreasing substance P in the dorsal root ganglia [10]. In addition, NMT can restore the tension of posterior urethral and bladder neck smooth muscle, reducing urine reflux and the risk of retrograde infection [28]. These pharmacological effects may be the main reason for the effectiveness of NMT against chronic epididymitis. Moreover, NMT combined with LVX further reduced CESI scores and epididymal nodule, with a response rate up to 97.22%, suggesting that the combination regimen could benefit more patients than either monotherapy regimen. Previous studies reported that NMT can inhibit the proliferation of *Staphylococcus* sp. and *Escherichia coli* and the formation of their biofilms [9, 29]. It is an important evidence that NMT combination therapy is superior to single-drug regimen by enhancing the sensitivity of bacteria to antibiotics and exerting a synergistic role. No adverse events or hepatorenal function indicators were observed during the study.

The present study has some limitations. The sample size was relatively small in this study, as it was designed to preliminarily observe the efficacy of NMT alone or combined with LVX in the treatment of chronic epididymitis. Therefore, the efficacy of NMT in the treatment of chronic epididymitis needs further verification with larger scale, multicentre randomized controlled trials. Additionally, the pharmacological mechanism of action of NMT remains to be further explored via biomolecular experiments.

In conclusion, this parallel randomized controlled clinical study showed that NMT could significantly improve CESI scores and epididymal nodule diameters in patients with chronic epididymitis. The combination of NMT and LVX provided a better effect than monotherapy and was well tolerated.

## Figures and Tables

**Table 1:** Patient demographics and characteristics at baseline (mean ± SD).

Variable	LVX (*n* = 36)		NMT (*n* = 40)		LVX + NMT (*n* = 36)		*P* value
	Mean ± SD	Range	Mean ± SD	Range	Mean ± SD	Range
Age (years)	36.00 ± 9.51	20∼58	41.28 ± 8.51	25∼59	40.92 ± 9.53	25∼58	0.7796
CESI	15.94 ± 1.87	12∼21	14.53 ± 2.97	8∼21	15.78 ± 2.26	13∼23	0.0520
MDE (cm)	1.34 ± 0.16	0.95∼1.60	1.30 ± 0.20	0.94∼1.72	1.28 ± 0.17	0.90∼1.67	0.3702

LVX, levofloxacin; NMT, Ningmitai; CESI, Chronic Epididymitis Symptom Index; MDE, mean diameter of epididymal nodules.

**Table 2:** CESI score after 2 or 4 weeks of treatment (mean ± SD).

Groups	LVX (*n* = 36)	NMT (*n* = 40)	LVX + NMT (*n* = 36)
CESI scores			
Baseline	15.94 ± 1.87	14.53 ± 2.97	15.78 ± 2.26
2 weeks	14.92 ± 1.66	13.33 ± 3.22^*∗*^	12.53 ± 2.64^*∗∗∗∗*^
4 weeks	10.39 ± 2.31	10.65 ± 3.49	8.64 ± 2.15^*∗∗*^^,##^

LVX, levofloxacin; NMT, Ningmitai. ^*∗*^, *P* < 0.05 compared with the LVX group. ^*∗∗*^, *P* < 0.01 compared with the LVX group. ^*∗∗∗∗*^, *P* < 0.0001 compared with the LVX group. ^##^*P* < 0.01 compared with the NMT group.

**Table 3:** Clinical efficacy rates after 2 and 4 weeks of treatment.

	2 weeks			4 weeks		
	LVX	NMT	LVX + NMT	LVX	NMT	LVX + NMT
*n*	36	40	36	36	40	36
Clinical efficacy, no. (%)						
Effective	3 (8.33)	22 (55.00)	22 (61.11)	30 (83.33)	32 (80.00)	35 (97.22)
Ineffective	33 (91.67)	18 (45.00)	14 (38.89)	6 (16.67)	8 (20.00)	1 (2.78)
*χ* ^2△^	—	18.69	22.12	—	0.1401	2.5320
*P* value^△^	—	<0.0001	<0.0001	—	0.7082	0.1116

LVX, levofloxacin; NMT, Ningmitai. ^△^Compared with the LVX group.

**Table 4:** Evaluation of hepatorenal function after 4 weeks of treatment.

	LVX	NMT	LVX + NMT	Reference range
*n*	36	40	36	
ALT (U·L^−1^)				
V0	29.94 ± 13.667	28.33 ± 10.406	32.72 ± 11.609	9∼50
V2	32.33 ± 12.205	31.88 ± 8.762	32.94 ± 10.559	
BUN (mmol·L^−1^)				
V0	4.78 ± 1.104	4.97 ± 0.933	5.43 ± 1.072	2.8∼7.14
V2	4.77 ± 1.072	5.13 ± 1.145	5.46 ± 1.065	
CREA (*μ*mol·L^−1^)				
V0	71.39 ± 20.080	69.50 ± 20.796	78.72 ± 20.928	40∼135
V2	72.03 ± 21.856	61.30 ± 17.164	81.98 ± 19.655	

LVX, levofloxacin; NMT, Ningmitai; ALT, alanine aminotransferase; BUN, blood urea nitrogen; CREA, creatinine.

## Data Availability

The data used to support the findings of this study are included within the article.
